# Comprehensive Mechanism of Gene Silencing and Its Role in Plant Growth and Development

**DOI:** 10.3389/fpls.2021.705249

**Published:** 2021-09-07

**Authors:** Ahmed H. El-Sappah, Kuan Yan, Qiulan Huang, Md. Monirul Islam, Quanzi Li, Yu Wang, Muhammad Sarwar Khan, Xianming Zhao, Reyazul Rouf Mir, Jia Li, Khaled A. El-Tarabily, Manzar Abbas

**Affiliations:** ^1^School of Agriculture, Forestry and Food Engineering, Yibin University, Yibin, China; ^2^Genetics Department, Faculty of Agriculture, Zagazig University, Zagazig, Egypt; ^3^Key Laboratory of Sichuan Province for Refining Sichuan Tea, Yibin, China; ^4^College of Tea Science, Yibin University, Yibin, China; ^5^College of Horticulture, Northwest A&F University, Xianyang, China; ^6^State Key Laboratory of Tree Genetics and Breeding, Chinese Academy of Forestry, Beijing, China; ^7^Research Institute of Forestry, Chinese Academy of Forestry, Beijing, China; ^8^Center of Agriculture Biochemistry and Biotechnology, University of Agriculture, Faisalabad, Pakistan; ^9^Division of Genetics and Plant Breeding, Faculty of Agriculture (FoA), Sher-e-Kashmir University of Agricultural Sciences and Technology (SKUAST–K), Sopore, India; ^10^Department of Biology, College of Science, United Arab Emirates University, Al-Ain, United Arab Emirates; ^11^Harry Butler Institute, Murdoch University, Murdoch, WA, Australia

**Keywords:** transcriptional gene silencing, post-transcriptional gene silencing, genomics imprinting, paramutation, RNAi, CRISPR/Cas9

## Abstract

Gene silencing is a negative feedback mechanism that regulates gene expression to define cell fate and also regulates metabolism and gene expression throughout the life of an organism. In plants, gene silencing occurs *via* transcriptional gene silencing (TGS) and post-transcriptional gene silencing (PTGS). TGS obscures transcription *via* the methylation of 5′ untranslated region (5′UTR), whereas PTGS causes the methylation of a coding region to result in transcript degradation. In this review, we summarized the history and molecular mechanisms of gene silencing and underlined its specific role in plant growth and crop production.

## Introduction

Gene silencing is a molecular mechanism that knocks down the gene expression in plants both in nature and in response to external stimuli (Wassenegger, 2002). It plays a pivotal role in plant defense mechanisms by detecting an aberrant RNA *via* nonsense-mediated messenger RNA (mRNA) decay (NMD), which could be fatal in case of remains in the RNA pool of the cell. Gene silencing mechanisms in plants are transcriptional gene silencing (TGS) and post-transcriptional gene silencing (PTGS) (Kelly and Aramayo, 2007). TGS is a nuclear-localized mechanism, which quenches transcription by blocking a promoter region for the binding of transcriptional machinery (Vaucheret and Fagard, 2001). Different methods of TGS are RNA-directed DNA methylation (RdDM), genomic imprinting, paramutation, transposon silencing, transgene silencing, and position effect. Notably, TGS is predominantly responsible for transposon and transgene silencing, but PTGS plays a limited role in this silencing (Wakimoto, 1998).

Post-transcriptional gene silencing is a cytoplasm-localized phenomenon to precisely target and degrade mRNA transcripts of specific genes (Vaucheret et al., 2001). Different methods of PTGS are RNA interference (RNAi), clustered regularly interspaced short palindromic repeats (CRISPR/Cas9), and NMD (Zebec et al., 2016). Recently, several studies have been conducted on gene silencing by deploying RNAi, the virus-induced gene silencing (VIGS), and CRISPR/Cas9 to enhance the resistance of plants against pathogens, drought tolerance, and lingo-cellulose pathway engineering (Borrelli et al., 2018; Abbas et al., 2020). In plants, small RNAs (sRNAs), such as microRNA (miRNA) and small-interfering RNA (siRNA) play a key role in plant fight against pathogens (Axtell, 2013). Gene silencing causes the periodic knock down of gene expression at the mRNA or protein level. Gene silencing spatiotemporally controls the regulation of gene networks, which subsequently regulate developmental processes in plant metabolism, such as genome stability, the detoxification of plant waste, and allergens (Mirouze et al., 2009; Ito, 2013).

Position variegation or position effect is a kind of gene silencing that was first discovered in *Drosophila melanogaster* by Muller (1930), which opened up new avenues of studies, such as genetics and functional genomics, and subsequently paved the way for the exploration of other possible gene silencing mechanisms in different organisms. After 1 year, a new type of gene silencing phenomenon was discovered in petunia, namely “co-suppression” (Napoli et al., 1990). VIGS was unexpectedly discovered in a hit and trial experiment (Ruiz et al., 1998). An mRNA sequence which encodes color pigments was artificially designed and ligated in a vector of virus origin, which resulted in the synthesis of double-stranded RNA (dsRNA) with its complementary counterpart on introduction in to petunia plant, dsRNA molecules subsequently triggered RNA induced gene silencing complex (RISC) which cleaved all dsRNA molecules of that specific gene and resulted in albino phenotype.

**FIGURE 1:**
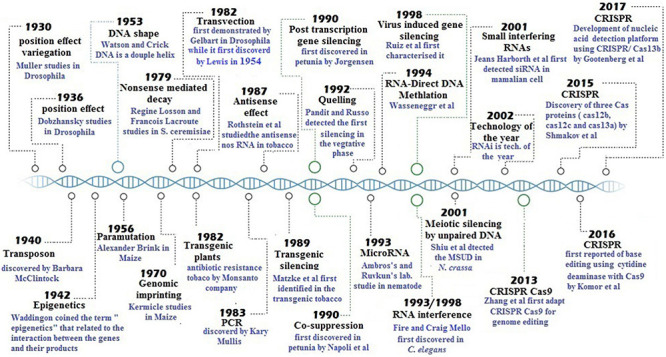
History of gene silencing.

A remarkable breakthrough in gene silencing research was the discovery of RNAi when Fire et al. (1998) introduced artificially designed single- and double-stranded *unc-22*-nt RNA molecules in *Caenorhabditis elegans* to observe phenotypic outcomes. As a surprise, albino phenotype was observed because both sense and antisense RNA strands were completely degraded (Fire et al., 1998). CRISPR is a robust gene silencing mechanism discovered in early 1993 by Francisco Mojica in prokaryotes (Nobel Prize) (Mojica et al., 2005), which was employed first time by Zhang et al. (2013) for genetic engineering in eukaryotes (Cong et al., 2013). A pictorial representation of the history of all silencing techniques has been illustrated in Figure 1. In this review, we comprehensively underlined the mechanism of gene silencing, molecular mechanisms behind gene silencing, and their pivotal roles in plant growth and crop production.

## Types of Gene Silencing

### Transcriptional Gene Silencing

#### RNA-Directed DNA Methylation

RNA-directed DNA methylation is a fundamental epigenetic gene silencing phenomenon almost found in all living organisms (Wassenegger et al., 1994). In RdDM, siRNAs transactivate the RISC of ∼21–24 nt, which regulates gene silencing *via* homologous DNA methylation. RISC is comprised of the following components: Argonaut (AGO) proteins, DNA methyltransferase (DNMT), chromatin remodeling complexes, and RNA polymerase IV-V (Mette et al., 2000; Sijen et al., 2001). The RdDM pathway is induced by the generation of dsRNA by transposable elements (TEs), transcribed inverted repeats (IR), viral replication intermediates, and RNA-directed RNA polymerase (RDR) (Mette et al., 2000; Sijen et al., 2001). DNA methylation predominantly occurs at the following nucleotide combinations of an RNA-DNA sequence homology: CG, CHG, and CHH (H is; A, C, or T) (Pélissier and Wassenegger, 2000). Both symmetric sequences, such as CG and CHG are methylated by methyltransferase 1 (MET1) and chromomethylase 3 (CMT3), whereas an asymmetric sequence of CHH is methylated by DNMTs, chromomethylase 2 (CMT2), and domains rearranged methyltransferase 2 (DRM2) (Huang and Zhu, 2014; Ito and Kakutani, 2014).

**FIGURE 2:**
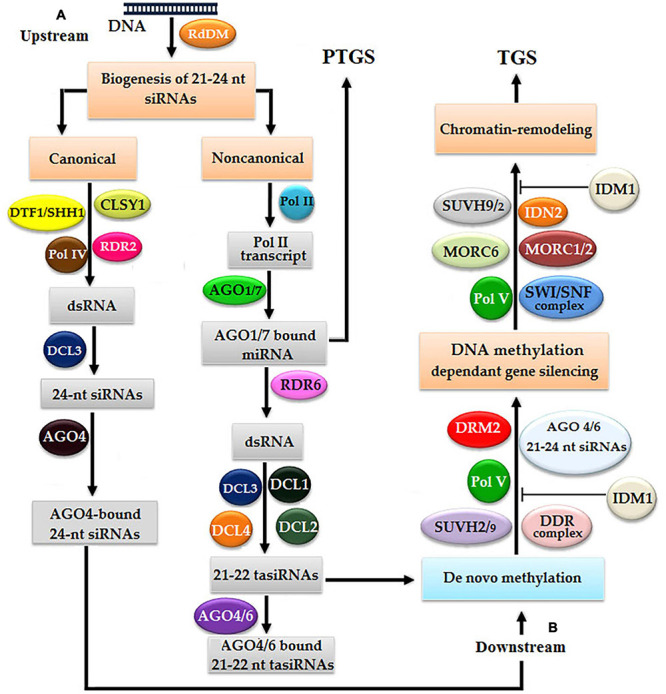
*Arabidopsis* models of RNA-directed DNA methylation (RdDM): double-stranded RNAs (dsRNAs) proceed into small-interfering RNAs (siRNAs) *via* canonical and non-canonical pathways. Both pathways undergo two steps: **(A)** in the first step, siRNAs are produced, which subsequently bind with argonaut (AGO) and **(B)** in the second step, DNA methylation leads to chromatin remodeling resulting in gene silencing *via* transcriptional gene silencing (TGS).

The RdDM pathway was originally discovered in *Arabidopsis thaliana*, which completes in two sequential steps: the biogenesis of ∼23–24 nt siRNAs and subsequent *de novo* methylation (Figure 2; Wierzbicki, 2012; Matzke et al., 2015). There are two types of pathways for the biogenesis of siRNAs: canonical and non-canonical, and the selection of either pathway is decided by polymerase IV, RDR2, and Dicer homolog 3 (DCL3). The initiation of the canonical pathway begins with the binding of DNA transcription factor 1/Sawadee homeodomain homolog 1 (DTF1/SHH1) to lysine 4 (K4) of non-methylated histone 3 (H3) and eventually causes the methylation of lysine 9 (K9), which stimulates the transcription of a specific DNA region due to an interaction between polymerase IV and RDR2 with the assistance of chromatin remodeling SNF2 domain-containing protein Classy 1 (CLSY1), and finally, the biogenesis of dsRNA is started (Enke et al., 2011; Haag et al., 2012; Du et al., 2014). By using the endonuclease activity of endo-ribonuclease DCL3, dsRNA molecules are cleaved to produce ∼23–24 nt siRNAs (Wu et al., 2012; Kanno et al., 2013). Approximately 23–24 nt mature siRNAs intercalate with AGO4 or AGO6 to induce methylation (Kanno et al., 2013).

In the non-canonical pathway, non-coding RNAs of the telomere-associated satellite (*TAS*) gene are transcribed by polymerase II, which subsequently bind with AGO1 or AGO7. Some *TAS* transcripts are cleaved by miRNAs, and one cleaved RNA is transformed into dsRNA by RNA-dependent RNA polymerase 6 (RDR6), which acts as a substrate of Dicer-like 4 (DCL4) to finally produce ∼21–22 nt transacting siRNAs (ta-siRNAs) (Kanno et al., 2013). Finally, if ta-siRNAs make a complex with AGO4 or AGO6, then the TGS pathway is activated, contrastingly, if ta-siRNAs make a complex with AGO1 or AGO7, then the PTGS pathway is activated (Pontier et al., 2012; Marí-Ordóñez et al., 2013; Nuthikattu et al., 2013; Panda and Slotkin, 2013).

DNA methyltransferase is responsible for the methylation of DNA, which acts as a switch on/off of gene expression (Harris et al., 2018), and depends upon polymerase V, AGO4 bound ∼21–24-nt RNA complex, DNMT, DRM2, SRA-domain-containing proteins SUVH1, SUVH2, SU(VAR)3-9, and several other proteins (Fischer et al., 2006). SUVH proteins are predominantly responsible for the establishment of a heterochromatin chromatic domain using H3K9me. *Arabidopsis* harbor 10 members of the SUVH gene family, of which SUVH1, SUVH2, and SUVH4 have non-unanimous effects on heterochromatic methylation. SUV39H1 is involved in H3K9me of heterochromatin and along with Sirtuin 1 (SIRT1) regulates facultative heterochromatin (Vaquero et al., 2007). SUV39H1 binds with DNMTs 1 and 3a with the help of its PHD-like and HP1beta motif to regulate both global epigenetic modifications, such as DNA methylation and histone methylation (François et al., 2003). SUVH1 and SUVH3 are involved in the methylation of euchromatin by binding with DNAj1 and DNAj2, and act as transcriptional antisilencing factors (Harris et al., 2018). SUVH2 plays a key role in gene silencing in *Arabidopsis* by the heterochromatin formation, any mutation in SUVH2 resulted in the hypomethylation of DNA at chromocenter heterochromatin and its overexpression causes ectopic heterochromatization, which also resulted in severe loss in growth (Fischer et al., 2006).

The initiation of methylation starts with the initiation of transcription of the target DNA locus with the binding of polymerase V and methyl-DNA-binding proteins SUVH2/9, where the role of SUVH2 is dominant over SUVH9 (Johnson et al., 2014; Liu et al., 2014). SUVHs are comprised of a DDR complex [defective in meristem silencing 3 (DMS3), dopamine receptor D1 (DRD1), and DRM2], which mediates polymerase V functionality, enhances DNA methylation, and quenches the target gene expression (Law et al., 2011). Finally, chromatin remodeling takes place by SUVH2/9, while in this study, the role of SUVH9 is significant in the inhibition of IDM1. SUVH9 also mediates with SWI/SNF-dependent chromatin remodeling and development of a microconidia MORC6 complex (Zhu et al., 2013). Thus, the silencing of a target gene is accomplished with the end of chromatin remodeling (Figure 2; Matzke and Mosher, 2014).

#### Genomic Imprinting

Genomic imprinting is an epigenetic phenomenon in which alleles of the same gene express divergently depending upon the parent of origin like in alternative splicing (Feng et al., 2010). Genomic imprinting may affect the inactivation of entire chromosomes, such as paternal X-chromosome in marsupials (Cooper et al., 1971). Based on the dominance, imprinted genes are of two types, such as maternally expressed imprinted genes (MEGs) and paternally expressed imprinted genes (PEGs) (Garnier et al., 2008; Köhler et al., 2012). In plants, all dominant imprinted genes are expressed only in the endosperm of flowering plants (García-Aguilar and Gillmor, 2015). During endosperm development, multiple nuclear divisions are followed by the formation of distinct mitotic domains, which determine the peripheral endosperm (PEN), micropylar endosperm (MCE), or chalazal endosperm (CZE) (Boisnard-Lorig et al., 2001; Stangeland et al., 2003). Noticeably, the imprinted genes in plants and animals are only 2% and predominantly express in the CZE endosperm (Gehring, 2013). For example, paternally inherited *Pr1* reciprocal kernel-color allele of maize displayed colorless or spotted kernels, whereas maternally inherited same allele displayed colored kernels.

**FIGURE 3:**
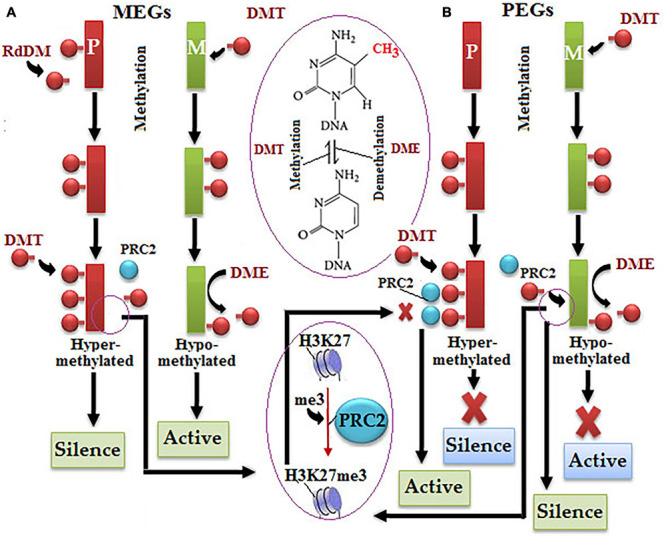
Molecular mechanism of alternative imprinting gene expression: **(A)** in the maternally expressed imprinted genes (MEGs) pathway, differential methylation of both maternal alleles occurs (green color), i.e., DEMETER (DME) downregulates DNA methylation by the hypomethylation process resulted in parental allele normal expression (red color), but increased methylation by DMT and polycomb repressive complex 2 (PRC2) causes histone methylation resulted in allele suppression, **(B)** in the paternally expressed imprinted genes (PEGs) pathway, the hypermethylated parental allele switches from the silent to active state under the action of PRC2 due to a halt in methylation by DMT, contrastingly hypomethylated maternal allele switches the active to silent state under the action of PRC2 *via* histone methylation.

Chromatin modifications have serious implications over the pattern of imprinted gene expression, such as methylated paternal allele remains transcriptionally silent in case of the demethylated maternal allele being transcriptionally active (Gehring et al., 2009). Genomic imprinting in *A. thaliana* got switched on due to a differential demethylation of DEMETER (DME) gene by DNA glycosylase, which dominantly expresses in female gametophyte (Choi et al., 2002; Schoft et al., 2011). The demethylation of DNA sequence repeats and TEs predominantly takes place by the removal of 5-methylcytosine. DMT is predominantly responsible for parental DNA methylation, and RdDM only occurs at MEG loci of parental allele (Figure 3; Köhler et al., 2012; Gehring, 2013; Zhang et al., 2013). H3K27me3 causes the suppression of the hypomethylated DNA of the maternal allele, but the polycomb repressive complex 2 (PRC2) interferes with the hypermethylated DNA of the parental allele at PGG to refrain it from the action of H3K27me3 resulted in the activation of the maternal allele (Figure 3; Weinhofer et al., 2010; Deleris et al., 2012; Makarevitch et al., 2013; Jermann et al., 2014).

#### Paramutation

Paramutation is an epigenetic phenomenon in which heritable changes in one allele are induced due to trans-interaction between two alleles at the same locus or different locus, which include DNA methylation and histone modifications (Chandler and Stam, 2004). The term “paramutation” was first coined by Alexander Brink to describe a puzzling phenomenon at the *r1* locus in maize (Brink, 1956). A positive paramutagenic allele is capable of transforming the second allele from a paramutable state to a new paramutagenic allele state (Chandler and Alleman, 2008). Paramutation is a kind of *trans*-regulation mechanism, which falls under the category of TGS (Hollick et al., 2000; Wagner et al., 2008). Noticeably, the mechanism of paramutation is similar to genetic recombination, transposition effect, and other genetic mutations (Hale et al., 2007).

**FIGURE 4:**
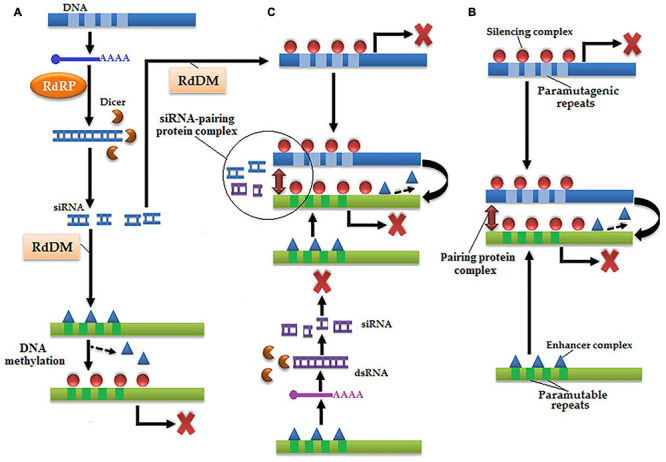
There are three models of paramutation: **(A)** RNA model, in which paramutagenic repeats of DNA are transcribed into dsRNA under the action of RdRP, which subsequently transformed into siRNA by a Dicer-like protein. Methylation of siRNA occurs *via* RdDM, which causes chromatin silencing of paramutable repeats by the addition of silencing complex inside enhancer complex resulted in the occurrence of paramutation. **(B)** Physical interaction, in which both paramutable and paramutagenic repeats physically interact by pairing protein complex and paramutation occurs epigenetically. **(C)** RNA-physical interaction, which is a combination of both abovementioned models, in which paramutable and paramutagenic repeats transcribed by RaRP into dsRNA, which are subsequently transformed into siRNA by a Dicer-like protein, and only cause chromatin silencing of paramutagenic sequence by RdDM. Subsequently, physical interaction is established *via* siRNA and pairing protein complex resulted in the modification of paramutable repeats.

Three models are used to describe paramutation: RNA model, physical interaction, and RNA–physical interaction (Figure 4; Stam, 2009). In the RNA model, only paramutagenic repeats are first transcribed into mRNA and then catalyzed by RdRP to transform into dsRNA, and finally dsRNA is cleaved by Dicer-like protein into siRNA (Grewal and Jia, 2007; Slotkin and Martienssen, 2007; Zaratiegui et al., 2007). These methylated free-state siRNAs are responsible for RdDM, which resulted in the silencing of a paramutable allele (Bühler et al., 2006). In the physical model, a physical interaction is established between both paramutable and paramutagenic alleles with the help of a pairing protein complex and transform paramutagenic alleles into paramutable alleles (Gohl et al., 2008). In the last combo-model, RdDM is accompanied by the physical interaction between paramutable and paramutagenic alleles.

#### Transposon Silencing

Transposable elements are auto-replicative short DNA repeats that can translocate within the genome (Sun et al., 2015). The main classes of TEs are DNA transposons and retrotransposons (RTs), which are further divided into two subclasses, such as autonomous and sub-autonomous (Feschotte et al., 2002). Autonomous TEs can translocate by themselves while non-autonomous TEs are dependent on other TEs for their translocation. The family of RTs is comprised of long terminal repeats-RTs (LTR-RTs) (class I), non-LTR-RTs (class II), short interspersed nuclear elements (SINEs), and pseudogenes. LTR-RTs are 100 bp to >5 kb long major internal coding repeats found in the genome of fungi, plants, and protists. LTR-RTs harbor reverse transcriptase, integrase, protease, RNase H, and capsid/gag proteins, which are inevitable for their transposition. Based on their encoded gene products and degree of sequence similarity, LTRs are of the following types: Ty1-copia RTs, Ty3-gypsy RTs, BEL/pao family, terminal repeat RTs in miniature (TRIMs), and endogenous retroviruses (ERV) (Havecker et al., 2004).

**FIGURE 5:**
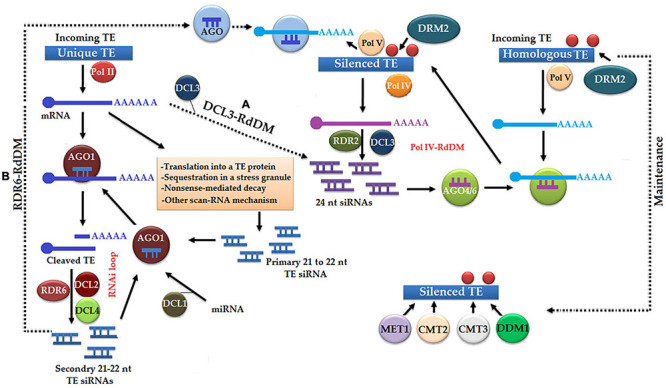
Homology-independent initiation of silencing is a primary pathway, which is unleashed with the expression of unique transposable elements (TEs) under the action of Pol II and messenger RNA (mRNA) undergoing post-transcriptional silencing *via* RNA interference (RNAi). In this pathway gene, silencing occurs by the following methods: **(A)** Dicer homolog 3- (DCL3-) RdDM, in which 24-nt siRNAs play a major role in a homology-dependent pathway, and **(B)** RNA dependent RNA polymerase 6- (RDR6-) RdDM, in which AGO protein-based silencing complex causes the silencing of Pol V transcripts. Homology-dependent pathway, in which TEs are transcribed by Pol IV and digested into 24-nt siRNAs by RDR2 and DCL3, which subsequently bind with AGO4 or AGO6 to become active and interact with polymerase V scaffolding transcript resulted in transcriptional TE silencing. Finally, silencing state is restored with the help of methyltransferase 1 (MET1), chromomethylase 2 (CMT2), chromomethylase 3 (CMT3), and DECREASE IN DNA METHYLATION 1 (DDM1).

The initiation of TE silencing occurs *via* the following two pathways, such as homology-dependent/identity-based and homology-independent/expression-based initiation of silencing (Figure 5; Fultz and Slotkin, 2017). In the upstream phase of a homology-dependent pathway, polymerase IV, RDR2, and DCL3 make a complex to produce 24-nt-long siRNAs from TEs associated with H3K9me (Nobuta et al., 2007; Huang et al., 2013; Law et al., 2013). In the downstream phase, a 24-nt-long siRNA molecule along with AGO4 or AGO6 protein interacts with polymerase V scaffold transcript resulting in transcriptional silencing of homologous TEs by the methylation of both DNA and H3K9me (Teixeira et al., 2009; Ito et al., 2011; Wierzbicki, 2012). Expression-dependent silencing of TEs is a kind of post-transcriptional silencing, which unleashes the synthesis of 21–22-nt-long siRNAs of target TEs with the help of miRNAs. Subsequently, the activation of the RNAi pathway helps in identifying and cleaving TE transcripts (Dunoyer et al., 2010; McCue et al., 2012).

#### Transgene Silencing

**FIGURE 6:**
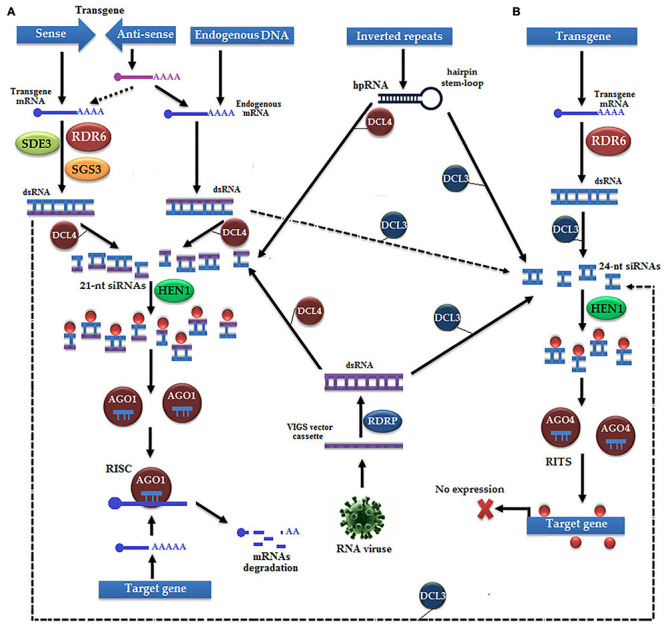
Transgene silencing occurs by the following two pathways: **(A)** post-transcriptional gene silencing (PTGS), which is initiated by sense or antisense transgenes, IR, hp-RNAs, and virus-induced gene silencing (VIGS). The sense strand is transcribed into mRNA, and transformed into dsRNA under the action of RDR6, suppressor of gene silencing 3 (SGS3), and RNA helicase (SDE3). Subsequently, dsRNA is either transformed into methylated 21-nt siRNAs by Dicer-like 4 (DCL4) and Hua Enhancer 1 (HEN1) or transformed into 24-nt siRNAs by AGO3 to cause gene silencing by the TGS pathway. The 21-nt siRNAs bind with AGO1 to make the RNA-induced gene silencing complex (RISC) that causes the degradation of mRNA of the target gene. Antisense strand is transcribed into mRNA, which either follows the same pathway with sense strand, or, like endogenous mRNA, transformed into dsRNA and bind with AGO to trigger the RISC. IR and VIGS follow either of the two available silencing pathways depending on the type of DCL. **(B)** TGS pathway, which is initiated with the transcription of transgene, dsRNA is formed under the action of RDR6, which is further cleaved into 24-nt siRNAs by DCL3, is methylated with HEN1, and make the RNA-induced transcriptional silencing (RITS) complex with the help of AGO4, which finally causes silencing.

Sometimes, the expression level of a successful transgene is not up to the mark due to: (a) the transgene insertion in heterochromatin or junk DNA (Tzfira et al., 2004) and (b) formation of complex T-DNA structures due to the binding of multiple integrated T-DNAs at a single locus (Gelvin, 2003). Transgene silencing occurs *via*: (a) PTGS triggered mechanisms by sense or antisense transgenes, IR, hairpin RNAs (hp-RNAs), and (b) VIGS (Figure 6; Wassenegger, 2002; Wang and Metzlaff, 2005). In transgene silencing, the first single-stranded sense RNA (ssRNA) becomes the dsRNA under the action of RDR6, RNA-binding suppressor of gene silencing 3 (SGS3), and RNA helicase (SDE3), and then cleaved by the RISC (Bond and Baulcombe, 2015). On the other hand, antisense ssRNA either directly hybridizes with endogenous ssRNA or indirectly hybridizes with the ssRNA of sense transgene to produce dsRNA to be finally cleaved by the RISC. Dicer-like enzymes (DCLs) determine the selection of pathways for the conversion of ssRNA to dsRNA, such as DCL3 being responsible for the selection of the TGS pathway and DCL4 for the PTGS pathway (Brodersen and Voinnet, 2006).

In the PTGS pathway, DCL4 performs an exonuclease activity to cleave dsRNA into 21-nt siRNAs, which are subsequently methylated by sRNA-specific methyltransferase Hua Enhancer 1 (HEN1) (Li et al., 2005). Methylated siRNA binds with AGO1 to make the RISC, which cleaves the entire mRNA of transgene and results in no phenotype or transgene silencing (Vazquez et al., 2004). Surprisingly, in some cases of transgene silencing, both sense and antisense strands undergo the TGS pathway under the action of DCL3 proteins, such as multicopy transgene loci, hp-RNA, and VIGS (Mishiba et al., 2005). The first mRNA of the transgene is converted into dsRNA with the help of RDR2 protein, cleaved into 24-nt siRNAs by DCL3, methylated by HEN1 to become capable of binding with AGO4 to make the RNA-induced transcriptional silencing (RITS) complex that finally causes DNA methylation of the transgene (Wassenegger, 2002; Wang and Metzlaff, 2005).

#### Position Effect

Position effect is a variation in gene expression due to translocation or inversion as a result of crossing over, chromosomal aberration, and transgene insertion (Weiler and Wakimoto, 1995). Position effect variegation (PEV) and telomeric position effect (TPE) are the two reasons of gene silencing during position effect. The reasons for gene silencing in PEV are the translocation of a gene from euchromatin to heterochromatin and vice versa (Girton and Johansen, 2008), modification in nucleosome by histone methylation, deacetylation, or structural changes (Bannister et al., 2001), and a close distance between gene and heterochromatin (Ryan and Vandenbergh, 2002). Gene silencing in TPE occurs when the transgene is inserted within or nearby a telomeric region (Pedram et al., 2006). Mosaic repeats, such as TAS-like sequences play a key role in gene silencing (Doheny et al., 2008). Gene silencing in mosaic repeats entirely depends upon histone modifications, such as standard tri-methylation H3K4, H3K9, and H4K20 (Boivin et al., 2003; Andreyeva et al., 2005).

**FIGURE 7:**
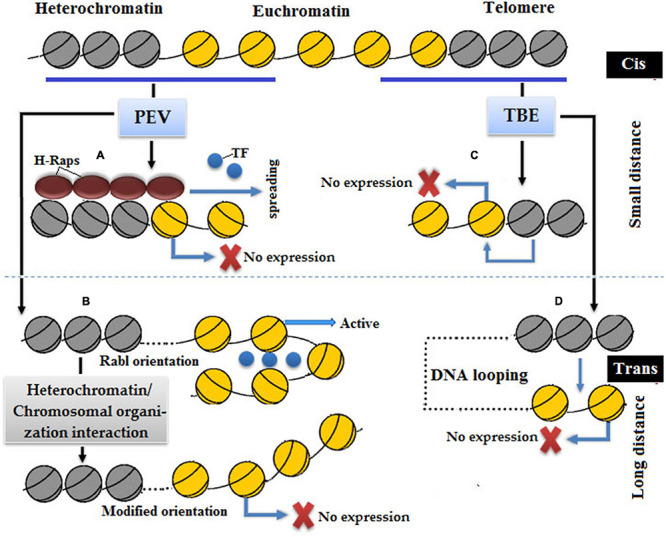
In position effect variegation (PEV) silencing: **(A)** cis-spreading model in which a repressor complex, namely, invading heterochromatin proteins H-Raps stop transcription by binding with a promoter of euchromatic gene. **(B)** Trans-interaction in which a new chromosomal orientation leads to silencing due to the loss of transcriptional machinery. In telomeric position effect (TPE) silencing, **(C)** cis-spreading model in which heterochromatin quenches adjacent genes resulted in gene silencing. **(D)** Trans-interaction in which distantly related euchromatin and heterochromatin come closer *via* DNA loop formation so that heterochromatin becomes capable of quenching the expression of euchromatin.

Epigenetic models that describe the molecular mechanism of PEV are cis-spreading and trans-interaction (Figure 7; Wakimoto, 1998). In the cis-spreading model, heterochromatin directly causes conformational changes in the packaging of euchromatin of transgene to refrain the binding of transcriptional machinery at a promoter resulted in transcriptional inhibition (Elgin, 1996). The cis-spreading model does not cover all aspects, such as some inserted genes induced variegation mode despite the long distance between the insertion site and breakpoint of heterochromatin (Henikoff and Dreesen, 1989; Weiler and Wakimoto, 1995). Trans-interactions or nuclear compartment is a comprehensive model to describe PEV, which means multiple interactions of different regions of heterochromatin and extensive chromosomal rearrangement resulted in gene silencing (Csink and Henikoff, 1996; Dernburg et al., 1996).

### Post-transcriptional Gene Silencing

#### RNA Interference

**FIGURE 8:**
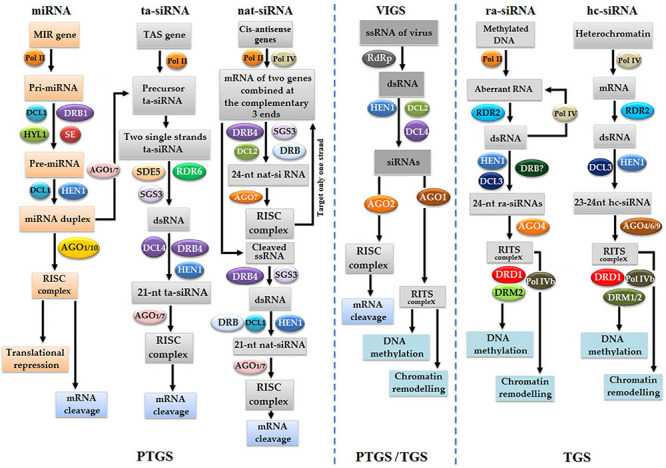
Naturally occurring gene silencing pathways in plants are PTGS gene silencing pathways, such as microRNA (miRNA), transacting siRNA (ta-siRNA), and natural-antisense siRNA (nat-siRNA), whereas TGS gene silencing pathways are repeated-associated siRNA (ra-siRNA) and heterochromatic siRNA (hc-siRNA). Meanwhile, VIGS is considered as artificial silencing methods that occurs *via* both TGS and PTGS pathways.

RNA interference is a homology-dependent gene silencing phenomenon that depends on dsRNA in gene silencing at the post-transcription level (Fire et al., 1998). The whole RNAi gene silencing factory consists of the following components: DCL, AGO, RDR, and dsRNA-binding domain (dsRBP) (Sabbione et al., 2019). sRNA biosynthesis is of primary importance in RNAi, which includes miRNAs and the following types of siRNAs: natural-antisense siRNA (nat-siRNA), ta-siRNA, heterochromatic siRNA (hc-siRNA), or repeated-associated siRNAs (ra-siRNAs) (Figure 8; Borges and Martienssen, 2015; Zheng et al., 2018). The cleavage of primary miRNAs (pri-miRNAs) into the precursor miRNAs (pre-miRNAs) is performed inside the nucleus under the action of DCL1, hyponastic leaves 1 (HYL1), and dsRBP. Then, pre-miRNA undergoes the second cleavage by DCL1 and HYL1 to finally produce miRNA duplex (Lu and Fedoroff, 2000; Vazquez et al., 2004). Subsequently, duplex miRNA is methylated by sRNA-specific methyltransferase HEN1 and exported to the cytoplasm with the help of exportin-5 ortholog HASTY (HST) (Park et al., 2002). Inside the cytoplasm, mature single-stranded miRNA binds with AGO1 to activate an RISC, which cleaves all homologous transcripts (Mallory and Vaucheret, 2006).

The second pathway of RNAi is dependent on the following long dsRNAs, nat-siRNA, ra-siRNA, ta-siRNA, and hc-siRNA, which could be exogenous due to viral infection or endogenous, such as transposons (Zheng et al., 2018). The nat-siRNA pathway is initiated with the transcription of natural-antisense gene pairs/overlapping genes distributed throughout the genome of plants. Overlapping genes are transcribed into two-three complementary mRNAs, which ligate each other to produce dsRNAs (Borsani et al., 2005). Subsequently, dsRNAs are cleaved by DCL2, SGS3, and RDR6 to generate 24-nt-long nat-siRNAs (Phillips et al., 2007), which bind AGO to activate the RISC to finally cleave out only cis-antisense mRNA strands. Trans-sense strand eventually becomes dsRNA under the action of RDR6 and SGS3, cleaved into 21-nt-long nat-siRNAs with the help of DCL1 and AGO1/7 to activate the RISC, and finally cleaves all homologous transcripts (Borsani et al., 2005).

Repeated-associated siRNAs and hc-siRNA are directly associated with methylation. In the ra-siRNA pathway, the promoter region of genes is methylated, so aberrant transcripts are produced with the help of RNA polymerase II, transformed into dsRNA under the action of RDR2, and follow further steps of gene silencing, or, dsRNA goes under the action of polymerase IVa to produce additionally aberrant RNA in a self-perpetuating loop (Xie et al., 2012). Subsequently, dsRNA is digested into 24-nt-long methylated ra-siRNAs under the action of DCL3 and HEN1, which binds with AGO4 to activate the (AGO4)-PolV complex or transcriptional silencing complex (RITS), causing the methylation of the complementary region of DNA with the help of SNF2-like chromatin remodeling proteins DRD1, DRM2, and alternative forms of polymerase Vb (Lippman et al., 2004; Xie et al., 2004). In the case of hc-siRNA, modified heterochromatin or DNA repeats are transcribed polymerase IV into mRNA, transformed into dsRNA under the action of RDR2, cleaved by DCL3 into 23–24-nt-long hc-siRNA (Chapman and Carrington, 2007; Matzke et al., 2009), binds with AGO6/9 to activate the RITC complex, and resulted in the TGS pathway (Chellappan et al., 2010; Fei et al., 2013). VIGS is another type of RNAi inbuilt defense system against viruses that can trigger both TGS and PTGS pathways of gene silencing in plants (Bartel, 2004; Burch-Smith et al., 2006; Lange, 2010).

#### Clustered Regularly Interspaced Short Palindromic Repeats/Cas9

**FIGURE 9:**
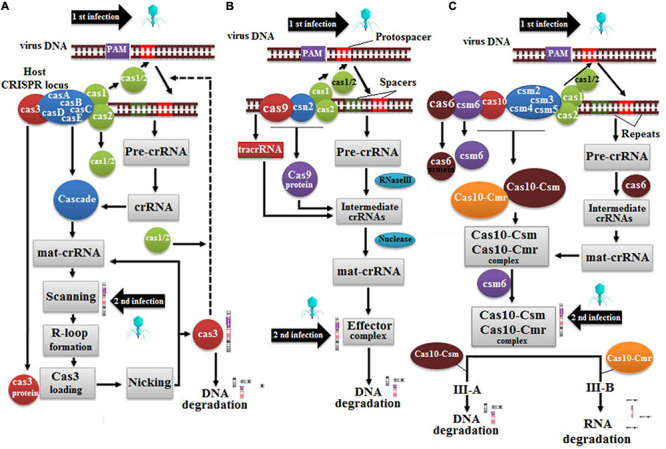
Cas protein mechanism is as follows: **(A)** Type I system depends on Cas3 protein, which recognizes new viral infection and counters it, **(B)** Type II system depends on Cas9 proteins and tracrRNA for the biogenesis of crRNA, and **(C)** Type III system depends on either Cas6 or Cas10 in which Csm targets DNA while Cmr targets viral RNA.

CRISPR is a natural immune system of Streptococcus pyogenes against viruses based on camera unit (Jinek et al., Science). A copy of invading virus genome is saved in bacterial geneome namely CRISPR array to deter future viral attack, and Cas9 protein cleaves out viral genome to refrain it from hijacking bacterial genome and cause disease (Makarova et al., 2011), and Cas9 protein cleaves out viral genome. The CRISPR/Cas system is further divided into Class I-III, and CRISPR/Cas9 lies under the umbrella of bacteria-specific Class II (Figure 9; Heler et al., 2015; Wei et al., 2015). Class I of the type II Cas system is further divided into three major types: I, III, and IV, whereas class II is further divided into the following major types: II, V, and VI. Type I CRISPR/Cas system is widely distributed among bacteria and archaea, which is subdivided into six subtypes (A to F) (Sinkunas et al., 2011). Cas3 has been amplified in all subtypes except A, B, and D, with a few variations in the protein sequence (Sinkunas et al., 2011). The second type of Cas system is comprised of Cas1, Cas2, and Cas9, furthermore, Cas2 belongs to type II-A (Barrangou et al., 2007; Heler et al., 2015) and Cas4 belongs to type II-B (Li et al., 2014). Noticeably, type I and type II Cas systems depend on the following two factors for gene editing, (a) CRISPR RNA (crRNA) spacer and (b) protospacer adjacent motif (PAM) (Mojica et al., 2009).

Generally, the CRISPR/Cas system is accomplished in three sequential steps: spacer acquisition, crRNA processing, and interference (Rath et al., 2015; Amitai and Sorek, 2016; Wang et al., 2016). On viral infection, Cas operon first expressed into a Cas1-Cas2 protein complex, which recognizes and makes a copy of protospacer of viral DNA, and integrates it between the repeated sequences of CRISPR array of the host genome. Subsequently, three CRISPR-related genes, trans-activating CRISPR RNA (tracr), and CRISPR array are transcribed into tracrRNA and pre-crRNA while the Cas9 gene encodes Cas9 protein. In the second step, tracrRNA anneals with the repeated sequence of pre-crRNA, and Cas9 protein binds with duplex. RNA transcript complex triggers RNase III enzyme to cleave pre-crRNA repeats (Jinek et al., 2012), nuclease finally produces the CRISPR/Cas9 complex by trimming extra nucleotides from 5́ end of pre-crRNA and leaving 20-nt-long spacer sequence (Liu et al., 2017). Finally, an interference step is kicked off, which activates the effector complex to recognize foreign DNA through its PAM site, such as 5́NGG3́. Subsequently, a spacer sequence of mature crRNA effector complex binds with the target sequence of viral DNA, which further activates RuvC and HNH domains of Cas9 protein (Gasiunas et al., 2012; Nishimasu et al., 2014). The RuvC domain cleaves non-target DNA strand, whereas the HNH domain cleaves the second strand to produce a blunt-end double-strand break in a 3-bp spacer region very next to the PAM site (Cong et al., 2013).

#### Nonsense-Mediated Decay

**FIGURE 10:**
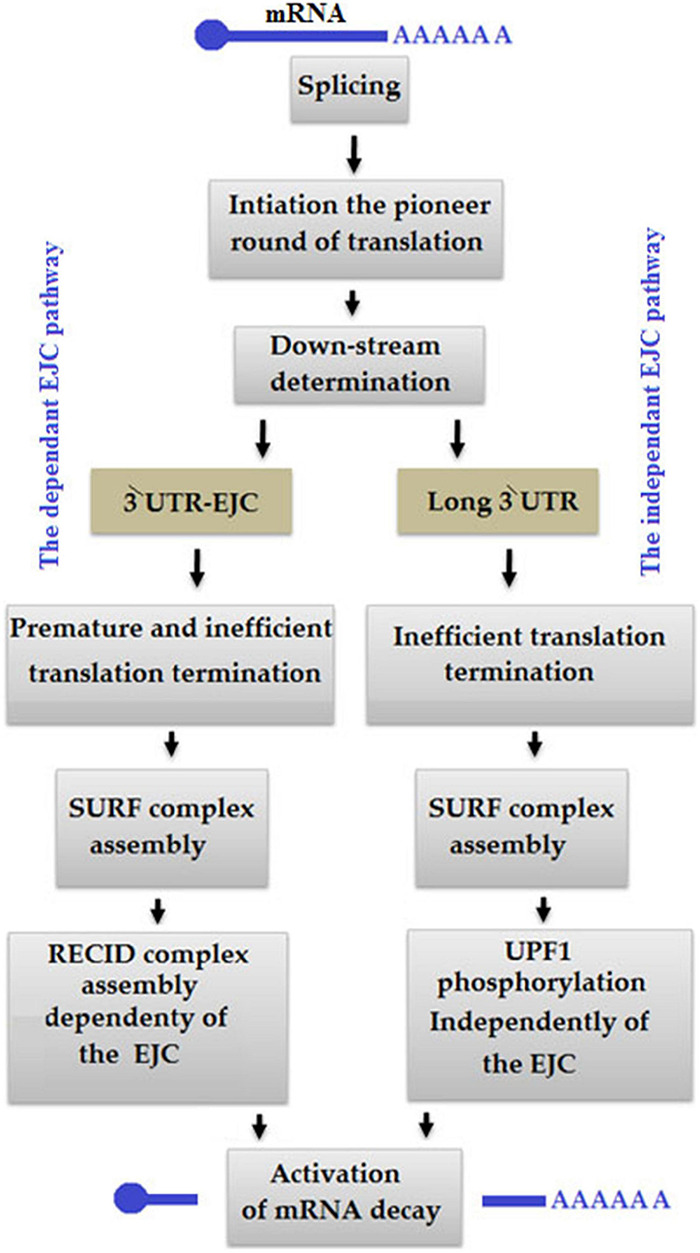
In an exon junction complex (EJC) pathway-dependent nonsense-mediated mRNA decay (NMD) model, the termination of translation occurs at the premature termination codon (PTC) level of ≥50–55 nt upstream of EJC, so that the ribosome is unable to dislocate it. In an EJC-independent pathway, PABPC1 distantly occurs from PTC to control eRF1-eRF3, which is related to the termination of translation.

Nonsense-mediated decay is considered as one of the most important RNA surveillance pathways, which occur at the post-transcriptional level (Gloggnitzer et al., 2014). NMD performs the two primary functions: (a) the regulation of transcription and (b) protein expression (Maquat, 2005), and remodel a gene product from proteins by targeting premature termination codons (PTCs) (Bhuvanagiri et al., 2010; Schweingruber et al., 2013; Miller and Pearce, 2014). Occasionally, abnormal translation during the NMD pathway occurs due to two reasons: (a) the ribosome is unable to bind with an exon junction complex (EJC) and (b) the ribosome removes the EJC (Figure 10; Kurosaki et al., 2014). At the same time, the PABPC1 is too distant from the PTC, so UPF1 usually combines with the termination complex leading to an independent NMD pathway (Amrani et al., 2004; Ivanov et al., 2008). EJC model is common in mammal cells, in which ≥50–55 nt upstream of the exon-exon junction a PTC exists while EJC is downstream of termination codon (Nagy and Maquat, 1998; Thermann et al., 1998).

In the NMD pathway, ribosome first binds with mRNA to initiate translation, removes existing EJC during the elongation of translation and stop at PTC, then eukaryotic release factors 1 and 3 of PABPC1 bind with the ribosome to make eRF1-eRF3 translation termination complex for translation termination (Kurosaki et al., 2014). Simultaneously, transient interaction between CBP80 and UPF1 enhances the attachment of serine/threonine kinase SMG1 complex to UPF1, which leads UPF1 to associate with eRF1 and eRF3 in the SURF complex (Kashima et al., 2006; Hwang et al., 2010). CBP80-UPF1 promotes SMG1-UPF1 binding to PTC-distal EJC in the DECID complex (Kashima et al., 2006). UPF1 is phosphorylated under the action of SMG1, which later causes mRNA decay and translational repression (Okada-Katsuhata et al., 2012; Durand et al., 2016).

## Roles of Gene Silencing in Plants

### Transgene Silencing

Genetic engineering is a promising technology for interspecies and intraspecies gene transfer to get desired traits, such as biotic and abiotic stress resistance, biofortification, higher yield in plants, but a serious constraint is transgene silencing (Stam et al., 1997). Gene silencing is a natural defense mechanism of living organisms against invaders including transgene (Rajeev Kumar et al., 2015). It was first observed in transgenic petunia transformed by chalcone synthase-A (CHS-A) gene, in which PTGS-directed gene silencing resulted in unexpected loss of flower pigments (Napoli et al., 1990). In particular, transgene silencing occurs *via* co-suppression, endogenous homology gene-induced PTGS pathway, and endogenous heterologous gene-induced PTGS pathway (Hamilton and Baulcombe, 1999). The PTGS pathway is initially induced in specific tissues and then transmit signals to nearby tissues, it does not only induce gene silencing in homologous transgenes but also in viral genes (VIGS) (Hamilton and Baulcombe, 1999). Furthermore, transgene along with a large number of intrinsic direct repeats significantly increases the frequency of induction of inheritable PTGS (Ma and Mitra, 2002).

### Role of Gene Silencing in Developmental Genes

The group of genes, which play a role in the developmental processes of plants, such as primary growth, secondary growth, meristem growth, leaf morphogenesis, secondary root elongation, organogenesis, and flowering, are called developmental genes. From the whole transcriptome, few numbers of coding mRNAs are translated into proteins to perform a specific function in a cell (Berget et al., 1977; Chow et al., 1977; Djebali et al., 2012). Contrastingly, non-coding mRNAs are undesired because they are unable to be translated into protein but occasionally encode sRNAs (Rajagopalan et al., 2006). The sRNAs target transcriptional and post-transcriptional silencing of developmental genes to maintain transcriptional equilibrium to enhance the adaptation of plant (Dunoyer et al., 2010). Natural RNAi, such as siRNAs and miRNAs, play a significant role in plant tissue development (Voinnet, 2009) by controlling the expression of *AGO1* and *DCL1* genes (Xie et al., 2003). Moreover, miR168 and AGO1-derived siRNAs participate in feedback mechanisms to regulate the expression of the *AGO1* gene (Mallory and Vaucheret, 2006; Rajagopalan et al., 2006). The regulation of gene expression in plants is performed by ta-siRNAs during the RNAi pathway (Peragine et al., 2004; Allen et al., 2005), by targeting mRNAs (Howell et al., 2007).

Genetic imprinting also affects the imprinting of developmental genes, such as angiosperms gene, which is involved in endosperm and seed size development (Bauer and Fischer, 2011; Haig, 2013). Genome-wide analysis of endosperm-related gene expression of rice revealed 162 MEGs and 95 PEGs relevant to imprinted differentially methylated loci, long non-coding RNAs (lncRNAs), miniature inverted-repeat TEs, and a few 21–22-nt-long siRNAs. One-half of PEGs and one-third of MEGs were related to nutrient metabolism and endosperm development, and thus represent grain yield quantitative trait loci (Yuan et al., 2017). Furthermore, few imprinted genes relevant to the transcription in endosperm cellularization and cell proliferation control seed size (Bauer and Fischer, 2011). Similarly, MEGs encoding transcription factor *OsMADS13* regulate the seed size in rice (Li et al., 2011), and PRC2 and AGL62 regulate the seed size in *Arabidopsis* (Kang et al., 2008; Lu et al., 2012). Furthermore, few TFs responsible for seed development in maize and *Arabidopsis* are also regulated under MEGs (Asano et al., 2002; Luo et al., 2005; Fu and Xue, 2010; Xin et al., 2013).

### Taming of TEs

Transposons or jumping genes are self-replicative short DNA sequences that can translocate within the genomes of the plant (Feschotte and Pritham, 2007; Sun et al., 2015). TEs are a severe threat to genome stability and are dealt with by TEs silencing (Kasschau et al., 2007; Slotkin and Martienssen, 2007), that is why a significant number of TEs remain silent in the plant genome (Okamoto and Hirochika, 2001). On the other hand, TEs can be beneficial in one population in the form of sRNAs (Ito et al., 2011; Parent et al., 2012), such as the activation of germinal cell RTs under stress stimulate the transcription of 24-nt-long siRNAs in *Arabidopsis* (Ito et al., 2011). TEs could be active or silent at a specific place and time within a genome, such as genes responsible for the silencing of pollen vegetative nuclei (VN) during seed development are downregulated, which were active during vegetative growth. Similarly, the final product of TEs from siRNAs enhances silencing mode in germ nuclei or sperm cell (SC) (Slotkin et al., 2009). Furthermore, TEs in double haploid rice plants cultured from anthers are reactivated after silencing as a result of somaclonal variations (Kikuchi et al., 2003). A relation exists between VN and SCs, which was observed in *Arabidopsis* and tobacco, causes the transcription of 21-nt-long siRNAs (McCormick, 2004).

### Gene Silencing Is a Key to Genomic Stability

Multiple factors influence genomic instability by DNA damaging or rearrangement, such as translocation and integration of TEs from one site to another within genome (Curcio and Derbyshire, 2003; Feschotte and Pritham, 2007). Transposons cause mutation within a genome, which are rectified under the PTGS pathway for genomic stability, and methylation also causes gene silencing resulted in a decrease in transposon activity. For example, the methylation of MET1 resulted in a decrease in PTGS activity and an increase in transposon activity (Ronemus et al., 1996; Morel et al., 2000; Kato et al., 2003), whereas a mutation in DECREASE IN DNA METHYLATION 1 (DDM1) causes a halt in a ratio of CG/non-CG methylation and increase the activity of transposons in *Arabidopsis* and *C. elegans* (Hirochika et al., 2000; Miura et al., 2001; Tsukahara et al., 2009). Similarly, three mutations *rde2, rde3*, and *mut7* in the PTGS pathway resulted in a higher activity in transposons in *C. elegans*, and *mut6* in *Chlamydomonas* also displayed the activation of transposons. In conclusion, TGS and PTGS pathways along with different siRNAs quench the activity of transposons to avoid genomic instability in plants (Mirouze et al., 2009; Ito, 2013).

### Detoxification of Toxins and Allergens in Plants

Plants are a major source of nutrition for all living organisms, but a few plant species harbor health-hazardous toxins and allergens that should be detoxified (Johansson et al., 2004; Lee and Burks, 2006). Furthermore, 90% of all food allergens are found in the following eight types of foods: soybean, peanut, wheat, tree nuts, fish, egg, shellfish, and milk (Zuidmeer et al., 2008), and to date, there is no proper treatment of food allergy except precautionary measures (Sicherer and Sampson, 2006). The PTGS mechanism of gene silencing, such as RNAi is pivotal to detoxify toxins and allergens in rice and soybean (Gu et al., 2016). In rice, antisense mRNA strategy of gene expression leads to a loss in a 14–16-kDa allergenic protein motif (Sheehy et al., 1988; Tada et al., 1996), while soybean harbors Gly-m-Bd-60K, Gly-m-Bd-30K, and Gly-m-Bd-28K seed-specific allergens (Ogawa et al., 2000), and Gly-m-Bd-30K (P34) was completely detoxified using transgene-induced gene silencing (Herman et al., 2003). RNAi is a super-duper technique against toxins and allergens, for example, the knock down of *7-Nmethylxanthine methyltransferase* gene (*CaMXMT1*) resulted in 70% reduced caffeine contents in transgenic plants (Ogita et al., 2003). Similarly, the knock down of cytochrome P450, CYP79D1, and CYP79D2 resulted in 90% loss in cyanogenic glucoside contents tubers of cassava (*Manihot esculenta*) (Siritunga and Sayre, 2003).

The downregulation of phytochelatin synthase gene (*OsPCS1*) in rice with the help of RNAi resulted in reduced accumulation of toxic metal Cd (Li et al., 2007), and nicotine demethylase gene knocks down in tobacco with the help of RNAi resulted in reduced nitrosamines [tobacco-specific N-nitrosamines (TSNAs)] carcinogen (Lewis et al., 2008). sRNAs successfully reduced terpenoid gossypol toxin contents in seeds and oil of cotton (Sunilkumar et al., 2006). Mal d1 allergen of apple (*Malus domestica*), Lyc e1 and Lyc e3 allergens of tomato, and Ara h2 allergen of peanut were significantly reduced with the help of RNAi (Gilissen et al., 2005; Le et al., 2006a, b; Dodo et al., 2008). The downregulation of the pathogenesis-related 10 (*PR 10*) gene with the help of RNAi resulted in decreased Dau c1.01 and Dau c1.02 allergens in patients (Peters et al., 2011).

### Regulation of Expression of the Endogenous Genes

Genes are always under a specific promoter that controls both the site and time of their expression, which is regulated by different kinds of gene silencing, i.e., transgene silencing. On exogenous or transgene expression, endogenous genes undergo non-symmetrical methylation by an RNA-chromatin mechanism resulted in the silencing of endogenous genes by the activation of the TGS pathway (Meyer et al., 1993; McGinnis et al., 2006). Furthermore, if a transgene induces the PTGS pathway, it can stop the expression of both exogenous and its homologous endogenous gene (Napoli et al., 1990; Van der Krol et al., 1990). The transgenic effect, in which both exogenous and endogenous genes are suppressed is known as co-suppression, which was observed in *Petunia* for the first time when *CHS-A* gene of pigmentation was overexpressed, which resulted in the suppression of both transgene and endogenous genes (Napoli et al., 1990).

Antisense and hp-RNA transgenes induced silencing in two endogenous genes in *Arabidopsis* resulted in more 4n than 2n plants due to a high level of methylation in tetraploid plants (Finn et al., 2011). The effect of gene silencing on the endogenous gene has also been observed in tobacco plants, in which the occurrence of co-suppression to nitrite reductase, nitrate reductase, and SAM synthase resulted in necrotic or chlorotic phenotypes (Palauqui et al., 1996). Comparatively, the transgene is more efficient against RNA silencing than endogenous genes in plants (Rajeev Kumar et al., 2015). RNAi-induced PTGS pathway of gene silencing also suppresses endogenous genes (Zhang et al., 2015). RNAi plays a significant role in silencing endogenous genes relevant to metabolic disease pathways, such as gluconeogenesis and phosphoenolpyruvate carboxykinase (PEPCK) enzyme (Zimmermann et al., 2006). PTGS enhances the gene silencing of endogenous genes, but bidirectional cytoplasmic RNA decay hinders its action on the endogenous gene (Zhang et al., 2015). NMD also plays a substantial role in the dynamic regulation of gene expression by controlling alternative splicing (Palusa and Reddy, 2010).

### Gene Silencing Provides Immunity Against Biotic Stress

Biotic stress factors exert serious implications on plants including viruses, bacteria, fungi, insects, and nematodes (Romanel et al., 2012). Gene silencing enhances the immunity of plants, which plays a pivotal role to counter biotic stress. Plant viruses seriously affect plant growth, which resulted in a significant loss in yield. Although plant viruses do not directly affect animals and humans, losses in food quality and crop yield are noted. In this study, RNA silencing plays a key role to deter viral genome integration in the host genome by cleavage and protects the plant against several viruses (Pumplin and Voinnet, 2013). RNA silencing depends on sRNAs, which are further subdivided into two classes: siRNAs and miRNAs (Wang and Smith, 2016). siRNA performs a preferred antiviral activity, which induces gene silencing by transitive siRNA and defensive signal, but its limitation is that it remains inactive until the infection begins (Eamens et al., 2008).

Contrastingly, the expression of miRNAs is constitutive and directly targets the viral genome on entry inside the host cell to assure plant protection while siRNA indirectly activates the biogenesis of 22–24-nt-long siRNAs, which subsequently respond to viral infection (Simón-Mateo and García, 2011). For example, miR156 and miR164 express P1/HC-Pro turnip mosaic virus—(TuMV-) encoded RNA silencing suppressors in *Arabidopsis* (Kasschau et al., 2003). Noticeably, artificially designed miRNA (amiRNAs) also induced resistance against grapevine virus A in tobacco (Roumi et al., 2012). Recently, CRISPR/Cas9 and CRISPR/Cas13a are being employed in enhancing the resistance against both RNA and DNA viruses by mutating susceptible genes in the host (Chandrasekaran et al., 2016).

Bacterial pathogens cause severe diseases in the targeted plant organs, such as scabs, leaf spots, and cankers, which can be controlled by gene silencing. For example, crown gall disease caused by *Agrobacterium* is being controlled by targeting silencing viz and iaaM genes by RNAi (Dunoyer et al., 2006). miRNAs and siRNAs play a pivotal role in defense against bacterial infection, such as the regulation of an auxin signaling pathway by miR393, resulted in enhancing plant antibacterial PTI (Navarro et al., 2006), and miR167, miR393, and miR160 play a role against bacteria in tomato (Fahlgren et al., 2016). NMD is a kind of PTGS gene silencing pathway, which contains bacterial infection by enhancing innate immunity of plants against bacterial infection by controlling numerous TIR domain-containing, nucleotide-binding, leucine-rich repeat (TNL) immune receptor-encoding mRNAs (Gloggnitzer et al., 2014). CRISPR/Cas9 and its roles in enhancing plant resistance against bacteria (Zaynab et al., 2020), such as CRISPR/Cas9-induced OsSWEET13 rice mutants displayed enhanced immunity against bacterial blight (Zhou et al., 2015).

Fungi cause 70% of the total plant diseases including smut, rusts, and mildew (Dean et al., 2012). Host-pathogen interaction at the surface of the host cell is established *via* haustorium and resulted in an exchange of signal and nutrients (Panstruga, 2003). Gene silencing enhances plant resistance against a broad range of fungal pathogens by the transfer of siRNAs or silencing signals from the host to the pathogen (Duan et al., 2012). For example, Avra10 in wheat and barley is a host-induced gene that limits fungal pathogen *Blumeria graminis* due to silent point mutations (Nowara et al., 2010). Similarly, the overexpression of miR1138 in wheat countered infection caused by *Puccinia graminis* (Gupta et al., 2012), and the downregulation of miR1448 and miR482 in cotton resulted in severe *Verticillium* infection (Jagadeeswaran et al., 2009). Moreover, the confirmed roles of some miRNAs-like *Md-miRln20* allow enhancing resistance in apple against Glomerella leaf spot (Zhang et al., 2019). CRISPR/Cas9 has a potential role in the activation of genes to confer the resistance against fungi, such as the transient expression of *TcNPR3 via* CRISPR/Cas9 resulted in conferring the resistance against *Phytophthora tropicalis in Theobroma cacao* (Fister et al., 2018).

RNA interference and CRISPR/Cas9 are being widely used in improving plant protection against insect pests, such as artificially designed dsRNAs that have been transformed to enhance plant resistance against Coleoptera and Lepidoptera (Price and Gatehouse, 2008). For example, the larval stage of cotton bollworm was reduced by transforming 22–24-nt-long artificially designed P450 monooxygenase genes, resulted in short feeding on plant tissues (Mao et al., 2007). Four wild populations of flour beetle (*Tribolium castaneum*) displayed that most of its variants harbor Cas9 target sites, and some of them are immune to drive and can be targeted (Drury et al., 2017). Nematodes also cause diseases and severe reduction of yield in many crops, which are being contained *via* gene silencing (El-Sappah et al., 2019). Approximately 30 miRNAs in *Arabidopsis* and 40 in soybean were differentially expressed during cyst nematodes infection (Hewezi et al., 2008), and miR159 plays a key role in gall and giant cell infection (Medina et al., 2017). RNAi is a robust technique for the development of plant resistance against nematode by reducing gall formation (Huang et al., 2006; Yadav et al., 2006).

### Gene Silencing Provides Immunity Against Abiotic Stress

An adverse effect of non-living surrounding factors on plants is known as abiotic stress, such as drought, heat, cold, light intensity, salinity, mineral deficiency, mineral toxicity, soil acidity, ozone, SO_2_, NO_2_, and higher CO_2_. Gene silencing *via* RNAi, CRISPR/Cas9, and miRNAs are promising to enhance plant resistance against the abovementioned abiotic stress conditions (Khraiwesh et al., 2012; Sun, 2012; Sunkar et al., 2012). Salinity is a critical abiotic factor significantly affecting crop productivity around the world (Bartels and Sunkar, 2005). Natural resistance, abiotic resistance genes, and miRNAs play a key role in plant tolerance against salinity (Ding and Zhu, 2009; Trindade et al., 2010). MiRNAs, miR159, miR160, miR167, miR169, miR393, and miR397 play a significant role in several plant species during salt stress (Sunkar and Zhu, 2004; Zhao et al., 2009; Gao et al., 2011; Kitazumi et al., 2015). During extended heat stress, miR398 plays a significant role in enhancing plant tolerance (Guan et al., 2013), whereas miR319 plays a significant role in extended cold tolerance (Thiebaut et al., 2019). In drought stress, miR160, miR167, miR169, and miR393 play a key role (Zhao et al., 2009; Sunkar, 2010), for example, miR171a, miR171b, and miR171c express in response to drought stress in potato (Hwang et al., 2011). MiRNAs regulate the cell wall to deter metal stress, for example, miR319, miR390, miR393, and miR398 express under Cu stress and miR160, miR164, and miR167 express under Cd stress (Yamasaki et al., 2007; Abdel-Ghany and Pilon, 2008; Huang et al., 2009) while miR390, miR168, miR156, miR162, miR166, and miR171 were downregulated and miR528 was upregulated (Ding et al., 2011).

### Improvement of Quality Traits

The quality traits of any crop are shape, color, shelf life, nutritional value, etc., which can be improved by conventional breeding and modern molecular techniques (Saurabh et al., 2014). Noticeably, the biggest challenge during the quality trait improvement is the loss of other desired characteristics, which can only be overcome by a selective transformation of flexible regulatory genes, potentially providing more competence and accurate regulation in a targeted manner (Tang and Chu, 2017). Gene silencing by deploying genetic engineering techniques, such as RNAi and CRISPR/Cas9 play a fundamental role in the regulation of genes relevant to quality traits in different crops (Saurabh et al., 2014). A miRNA is also a promising tool for the improvement of quality traits in different crops at the post-transcriptional level, such as miR156 and miR397 control grain size, quality, and yield (Jiao et al., 2010; Si et al., 2016), miR159 regulates stem elongation and floral development (Tsuji et al., 2006), and miR160 plays a critical role in the growth, development, and immunity of rice (Li et al., 2014). miR164 plays a key role in lateral root development, and miR166 in leaf polarity in maize (Juarez et al., 2004; Li et al., 2012), whereas miR159 plays an important role in anthers development and heat response in wheat (Wang et al., 2012). miR172 plays an essential role in cleistogamous flowering and grain density in barley (Nair et al., 2010; Houston et al., 2013). miR156 regulates vegetative and reproductive growth (Silva et al., 2014), whereas miR159, miR167, and miR4376 regulate flowering in tomatoes (Buxdorf et al., 2010; Wang et al., 2011; Liu et al., 2014). Recently, CRISPR/Cas9 has emerged as a promising tool for the identification of new genes, and genetic modification to improve quality traits and yield (Wang et al., 2019). CRISPR/Cas9 was used to improve fruit size by regulating classical CLAVATA-WUSCHEL (CLV-WUS) stem cell circuit (Ma et al., 2015), and the identification of new genes relevant to malate contents and aluminum-activated malate transporter 9 (ALMT9) in tomatoes (Ye et al., 2017).

### Gene Silencing Facilitates Functional Genomics

Tools in molecular biology have been developed for the identification, amplification, interspecies, and intraspecies transformation of desired genes, which predominantly rely on T-DNA activation and knockout libraries (Weigel et al., 2000). Comparatively, VIGS is efficient out of all available tools for the study of functional genomics (Cakir et al., 2010), due to (a) fast, (b) easy designing due to the independence of full-length cDNA, (c) transient gene silencing, (d) higher efficacy even in polyploid species, and (e) easy delivery (Cakir et al., 2010). RNAi is also being widely employed in studying functional genomics in many organisms without any limitation (Whitehurst et al., 2007; Smith et al., 2010). In plants, a lot of studies have been conducted with the help of RNAi to determine the function of different genes to improve plant resistance against biotic and abiotic stress, biofortification, lingo-cellulosic pathway engineering, and the improvement of quality traits. Furthermore, the discovery of CRISPR/Cas9 has revolutionized functional genomics exponentially (Liu et al., 2017).

## Advantages and Disadvantages of Gene Silencing

### Advantages of Gene Silencing

Transposons translocate within the genome and pose severe threats to genomic stability, which are deterred by gene silencing (Slotkin and Martienssen, 2007). Gene silencing maintains the balance of transcripts to ensure the adaptation of a plant to environmental fluctuations (Dunoyer et al., 2010). Tissue development and finishing are entirely controlled by gene silencing (ti-siRNAs and miRNAs) *via* a negative feedback mechanism (Voinnet, 2009; Cheng et al., 2021). Gene silencing plays a key role in crop yield by controlling the expression of genes related to seed size, for example, the silencing of *OsMADS13*, and PRC2 and AGL62 regulate seed size in rice (Li et al., 2011) and *Arabidopsis*, respectively (Kang et al., 2008; Lu et al., 2012). Gene silencing plays a key role to deter pathogenicity caused by biotic stress factors, such as insects, nematodes, bacteria, viruses, and fungi, which cause severe loss in crop yield (Zaynab et al., 2020). Allergy is an incurable disease mainly caused by the ingestion of daily food items, and gene silencing is helpful in the detoxification of these allergens (Gu et al., 2016). Caffeine contents were decreased by 70% in tea by the silencing of the *CaMXMT1* gene, cyanogenic contents were decreased by 90% by the silencing of cytochrome P450, CYP79D1, and CYP79D2 in cassava tubers (Ogita et al., 2003; Siritunga and Sayre, 2003), the silencing of *OsPCS1* in rice resulted in a significant loss in toxic metal contents (Li et al., 2007), and nicotine demethylase gene knockdown in tobacco resulted in reduced carcinogen (Lewis et al., 2008). Gene silencing also downregulates toxins in the seeds and oil of cotton, in apples, tomatoes, and peanuts.

### Disadvantages of Gene Silencing

To achieve higher crop yield, the scientist had developed genetic transformation techniques to overcome biotic and abiotic stress factors. Gene silencing negatively affects genetic transformation by the silencing of transgenes (Stam et al., 1997), such as the CHS-A gene, which was transformed in petunia to get a dark brown color but albino phenotype was observed in transgenic plants (Napoli et al., 1990). RTs are responsible for the activation of germinal cells in *Arabidopsis*, which goes silent due to gene silencing and results in the loss of switch from vegetative to reproductive growth (Ito et al., 2011).

## Author Contributions

AE-S, MA, KE-T, JL, and XZ: conceptualization. AE-S: writing the original draft and drawing figures. MI, KY, YW, RM, MK, JL, MA, XZ, AE-S, KE-T, QH, and QL: editing and proofreading. AE-S and MA: writing the final manuscript. All the authors reviewed and approved the final submission.

## Conflict of Interest

The authors declare that the research was conducted in the absence of any commercial or financial relationships that could be construed as a potential conflict of interest.

## Publisher’s Note

All claims expressed in this article are solely those of the authors and do not necessarily represent those of their affiliated organizations, or those of the publisher, the editors and the reviewers. Any product that may be evaluated in this article, or claim that may be made by its manufacturer, is not guaranteed or endorsed by the publisher.
